# Reversal of the Pinning Direction in the Synthetic Spin Valve with a NiFeCr Seed Layer

**DOI:** 10.3390/nano12122077

**Published:** 2022-06-16

**Authors:** Shaohua Yan, Weibin Chen, Zitong Zhou, Zhi Li, Zhiqiang Cao, Shiyang Lu, Dapeng Zhu, Weisheng Zhao, Qunwen Leng

**Affiliations:** 1 School of Integrated Circuit Science and Engineering, Beihang University, Beijing 100191, China; yanshaohua@buaa.edu.cn (S.Y.); zzt0807@buaa.edu.cn (Z.Z.); zhi.li@buaa.edu.cn (Z.L.); zhiqiangcao@buaa.edu.cn (Z.C.); zhudp@buaa.edu.cn (D.Z.); 2 Beihang-Goertek Joint Microelectronics Institute, Qingdao Research Institute, Beihang University, Qingdao 266100, China; lusy@bhqditi.com; 3 School of Physics, State Key Laboratory of Crystal Materials, Shandong University, Jinan 250100, China; wbchen@mail.sdu.edu.cn

**Keywords:** seed layer, exchange coupling, pinning direction

## Abstract

The effect of the seed layers on the magnetic properties of the giant magnetoresistance thin films has received a lot of attention. Here, a synthetic spin valve film stack with a wedge-shaped NiFeCr seed layer is deposited and annealed following a zero-field cooling procedure. The film crystallinity and magnetic properties are studied as a function of the NiFeCr seed layer thickness. It is found that the exchange coupling field from the IrMn/CoFe interface and the antiferromagnetic coupling field in the synthetic antiferromagnet both increase as the seed layer thickness increases, indicating the perfection of film texture. In this film, the critical thickness of the NiFeCr seed layer for the formation of the ordered IrMn_3_ texture is about 9.3 nm. Meanwhile, a reversal of the pinning direction in the film is observed at this critical thickness of NiFeCr. This phenomenon can be explained in a free energy model by the competition effect between the exchange coupling and the interlayer coupling during the annealing process.

## 1. Introduction

Spin valve structures have played an important role in the practical applications of the giant magnetoresistance (GMR) effect, such as magnetic random access memory, hard-disk read heads, biosensors, and e-compass [1,2,3,4]. The standard spin valve structure consists of two ferromagnetic (FM) layers separated by a conductive spacer layer. One of the FM layers is the so-called the free layer, which is of soft magnetic properties and can switch freely under an external magnetic field. The another one is the reference layer, whose magnetization direction is strongly pinned by the exchange bias effect to the adjacent antiferromagnetic (AFM) layer. In practice, a synthetic antiferromagnetic (SAF) structure is usually employed as the reference layer in the spin valve to improve its thermal stability and generate strong exchange coupling strength [5,6]. IrMn is the most commonly used antiferromagnetic material in such synthetic spin valves due to its high thermal stability, good corrosion resistance, and large exchange bias field [7]. Previous experimental studies show that the exchange bias effect is generally correlated with the (111) texture in the IrMn AFM layer [8,9]. A seed layer can affect not only the crystalline textural structure of the IrMn but also the performance of the layers deposited subsequently. Therefore, the appropriate choice of the seed layer is crucial for the textured growth of the thin films and the improvement of their performance.

Different types of seed layers have been explored in earlier studies, such as Ta, NiFe, Ru, NiCr, and NiFeCr [10,11,12,13,14,15,16,17]. Among them, NiFeCr attracts a great deal of attention. It has been reported that using NiFeCr as a seed layer can significantly enhance the magnetoresistance (MR) ratio and increase the pinning field, since NiFeCr can induce a strong (111) texture in the layers deposited subsequently [18]. The NiFeCr seed layer also exhibits a relatively high resistivity, which is beneficial for minimizing the current shunting effect in current-in-plane GMR devices [15,19]. Meanwhile, the NiFeCr layer can accommodate the Mn atoms and thus suppress the diffusion of Mn atoms into the magnetic layers to some extent. Therefore, NiFeCr is a promising candidate for the seed layer in the spin valve with Mn-alloyed antiferromagnetic materials [20].

In this work, a synthetic spin valve thin film stack with a wedge-shaped NiFeCr seed layer is fabricated. The structural and magnetic properties of the film are investigated. An unexpected reversal of the pinning direction is identified after annealing in the sample with a specific seed layer thickness. A macroscopic free energy model based on the single-domain assumption is established to explain the dependence of pinning direction on the seed layer thickness. At a high annealing temperature, the competition between the exchange coupling effect at the IrMn/CoFe interface and the interlayer coupling effect between the free layer and the reference layer plays a key role in determining the final pinning direction. The experimental results suggest that there is a critical seed layer thickness for the texture growth of the IrMn layer, which influences the stability of the pinning effect.

## 2. Materials and Methods

**Figure 1:**
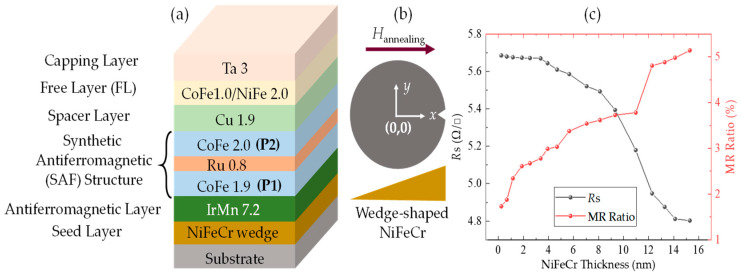
(**a**) Schematic of the film stack, (**b**) schematic of the wedge-shaped seed layer on the wafer, (**c**) sheet resistance and MR ratio of the film versus NiFeCr thickness.

The thin film stack with a structure of Ta(0.3)/Ni_47_Fe_11_Cr_42_ (wedge)/Ir_20_Mn_80_(7.2)/Co_70_Fe_30_(1.9)/Ru(0.8)/Co_70_Fe_30_(2.0)/Cu(1.9)/Co_70_Fe_30_(1.0)/Ni_81_Fe_19_(2.0)/Ta(3) (thickness in nanometer) is deposited on an 8-inch Si/SiO_2_ substrate by the Singulus Rotaris sputtering system under a base pressure of less than 4 × 10^−8^ torr. The NiFeCr seed layer thickness in this thin film ranges from 0 to 16 nm, as shown schematically in Figure 1a,b. The film is annealed for 1 h at 270 °C with a 10,000 Oe magnetic field along the +*x* axis, then cooled down without the field. The magnetic and electrical properties of the film are characterized at room temperature by a MicroSense KerrMapper. The measurement of magnetization loops is based on the longitudinal magneto-optical (MOKE) effect. The polarization change of the laser beam can be detected and analyzed by the machine, giving the results of Kerr rotation, which is related to the magnetization of the film. The sheet resistance of the film is measured based on the four-probe method. The KerrMapper is equipped with a probe card. During the measurement, four equally-spaced colinear probes are in contact with the surface film. A DC current of 1 mA is applied between the outer two probes, and the voltage is measured between the inner two probes. The structural analysis of the film is performed by means of X-ray diffraction (XRD) in which the diffraction angle is varied in the 2θ = 10°~80° range with Cu-Kα radiation.

## 3. Results

Figure 1c depicts the dependence of the sheet resistance *R*_S_ and MR ratio as a function of the NiFeCr seed layer thickness. The sheet resistance shows a monotonic decrease with the increase in the total metallic film thickness. The MR ratio, which is calculated as (R_max_ − R_min_)/R_min_, tends to increase with the seed layer thickness. The large sheet resistance with a thin seed layer is probably caused by the discontinuous and growth defects in the film. Such defects would enhance the electron scattering and thereby increase the sheet resistance [15].

**Figure 2:**
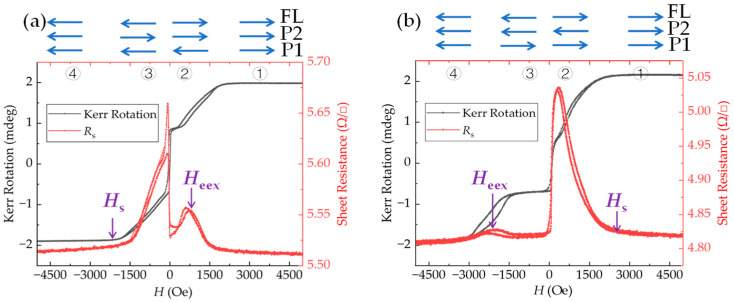
Magnetization loops (black line, left *Y*-axis) and *R*_s_-*H* loops (red line, right *Y*-axis) of the film with different NiFeCr thicknesses: (**a**) *t*_NiFeCr_ = 8.2 nm; (**b**) *t*_NiFeCr_ = 15.3 nm.

Figure 2 shows the longitudinal MOKE magnetization loops and the sheet resistance loops of the annealed film for different seed layer thicknesses. The blue arrows denote the magnetization directions of the three magnetic layers in the film stack (i.e., the free layer (noted as FL) and the two pinned layers in the SAF structure (noted as P1 and P2)). Four magnetization states are shown in the figures. For states 1 and 4, the magnetizations of the three layers are parallel due to the high applied field. As the field direction switches from positive (state 2) to negative (state 3), the magnetization of FL reverses, while the magnetizations of P1 and P2 remain fixed.

From these curves, two types of exchange coupling can be characterized by two characteristic fields. One is the effective exchange coupling field *H***_ee_**_x_, which is determined by the shift of the minor loop away from the zero magnetic field [21]. *H*_eex_ refers to the magnetic field needed to rotate the P1 magnetization by breaking both the exchange coupling from IrMn and the antiferromagnetic coupling from P2. The other one is the saturation field *H*_s_, which is the field required to saturate both magnetizations of the ferromagnetic layers in an SAF structure. This field is determined by the antiferromagnetic coupling strength in the trilayered CoFe (P1)-Ru-CoFe (P2) structure. By comparing Figure 2a,b, it is clear that the pinning directions are different in the film with different NiFeCr thicknesses. With a thick seed layer, the pinning direction (i.e., the magnetization direction of P1 layer) is along the +*x* axis and is consistent with that of the magnetic field applied during the annealing process. With a thin seed layer, the pinning direction is opposite to the former one.

**Figure 3:**
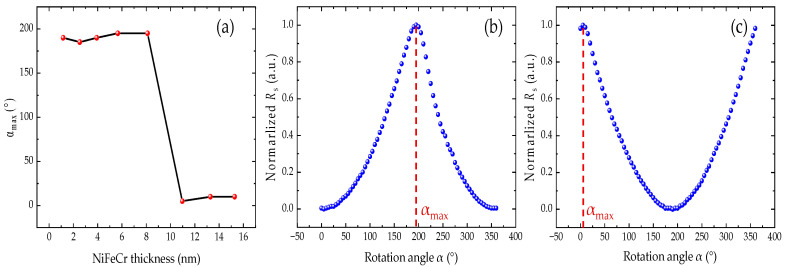
(**a**) Pinning direction variation as a function of the NiFeCr thickness; (**b**,**c**) angular-dependent sheet resistances at different NiFeCr thicknesses: (**b**) t = 8.2 nm and (**c**) t = 15.3 nm.

In order to verify the exact pinning directions at different seed layer thicknesses, the angular dependence of the sheet resistance of the film is measured, as illustrated in Figure 3. An external magnetic field of 30 Oe is applied for the angular dependence experiments, which is sufficient to rotate the FL magnetization but too small to rotate that of the SAF structure. The rotational angle α is the angle between the external field direction and the +*x* axis. The resistance of the synthetic spin valve is varied with the angle between the magnetizations of FL and P2. When the resistance reaches its maximum, the FL magnetization direction is antiparallel to that of the P2 and parallel to the magnetization direction of P1 (i.e., the pinning direction). We define this angle as α_max_. As summarized in Figure 3a, as the seed layer thickness increases to 11 nm, the pinning direction in the film is suddenly reversed, and α_max_ changes from around 180° (along the −*x* axis) to around 0° (along the +*x* axis).

**Figure 4:**
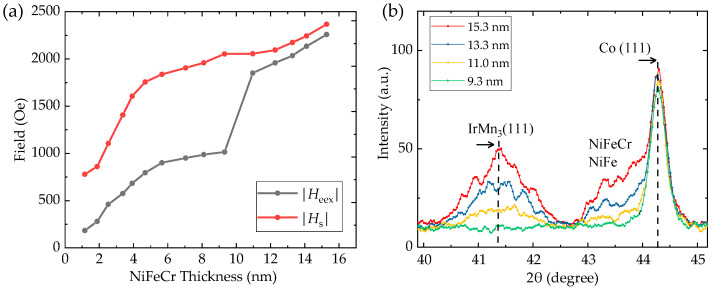
(**a**) Evolution of *H*_eex_ and *H*_s_ (in absolute values) with NiFeCr thickness; (**b**) intensities of IrMn_3_ (111) peak in the film with different seed layer thicknesses.

The magnetic parameters of *H*_eex_ and *H*_s_ derived from the magnetization loops are illustrated in Figure 4a. Both the absolute values of the *H*_eex_ and *H*_s_ increase as the NiFeCr thickness increases. When the thickness of the seed layer is larger than 9.3 nm, the *H*_eex_ value has a dramatic increase. Meanwhile, the pinning direction comes along the +*x* axis, opposite to that with the NiFeCr seed layer less than this thickness. The crystallographic characterization of the thin films is performed at room temperature by the XRD technique. As the (111) texture is indispensable for high GMR effects [22], only a portion of the scan including (111) peaks is shown in Figure 4b. The left peaks in the vicinity of 2θ ≈ 41.3° correspond to the ordered L1_2_-IrMn_3_ cubic (111) reflection [10]. A critical thickness effect can be found at a NiFeCr thickness of 9.3 nm for the film annealed at 270°, below which this (111) reflection peak becomes undetectable. The evolutions of *H*_eex_, *H*_s_, and MR ratio are related to the enhancement of the (111) IrMn texture, which also promotes a well-oriented texture of the layers deposited on it. It can be noticed that with the increase in seed layer thickness, the full width at half maximum intensity of this peak is slightly increasing, implying a smaller grain size according to the Scherrer equation. In a recent theoretical simulation work, the grain size dependence of the exchange bias is explained by the reduction in the statistical imbalance in the number of interfacial spins for large grains [23,24]. The plateau-like diffraction observed at 2θ = 42.7°–44° originates from a composite of (111) peaks of the NiFeCr seed layer and the NiFe layer. The peaks on the right correspond to the reflection of the fcc-Co (44.3°) structure.

## 4. Discussion

To shed light on the reversal of the pinning direction at the critical thickness, a free energy model based on the single-domain assumption to calculate the pinning direction during the annealing process is presented. The total energy of the SAF structure is mainly composed of the uniaxial anisotropy energies *E*_ani_ of P2 and P1, the interlayer coupling energy *E*_in_ between FL and P2, the RKKY coupling energy *E*_RKKY_ between P2 and P1, and the exchange coupling energy *E*_ex_ between P1 and the antiferromagnetic layer [6,25,26,27]. The total free energy *E*_total_ can be written as

*E*_total_ = *E*_ani_ + *E*_in_ + *E*_RKKY_ + *E*_ex_ (1)

*E*_ani_ = *H*_K2_ × *M*_P2_ × *t*_P2_ × sin^2^(*θ*_P2_)/2 *+ H*_K1_ × *M*_P1_ × *t*_P1_ × sin^2^(*θ*_P1_)/2 (2)

*E*_in_ = −*H*_in_ × *M*_P2_ × *t*_P2_ × cos(*θ*_P2_ − *θ*_FL_) (3)

*E*_RKKY_ = *H*_saf_ × *M*_P1_ × *t*_P1_ × *M*_P2_ × *t*_P2_ × cos(*θ*_P2_ − *θ*_P1_)/(*M*_P1_ × *t*_P1_ + *M*_P2_ × *t*_P2_) (4)

*E*_ex_ = −*H*_ex_ × *M*_P1_ × *t*_P1_ × cos(*θ*_P1_) (5)

Here, *M*_P1_ and *M*_P2_ are the saturated magnetization magnitudes of P1 and P2, and *t*_P1_ and *t*_P2_ are the thicknesses of the two ferromagnetic layers. The parameters in the above equations are given based on measurement values with *M*_P1_ × *t*_P1_ = 1.49 × 10^−4^ emu/cm^2^ and *M*_P2_ × *t*_P2_ = 1.8 × 10^−4^ emu/cm^2^. *H*_K1_ and *H*_K2_ are the induced uniaxial anisotropy fields of P1 and P2. During the calculation, *H*_K1_ and *H*_K2_ are set to be 30 Oe, a typical value for 2 nm CoFe [28]. *θ*_P1_, *θ*_P2_, and *θ*_FL_ are the angles between the magnetizations of P1, P2, and FL and the +*x* axis, respectively. *H*_in_ is the interlayer coupling field between the FL and P2 layers. *H*_saf_ is the antiferromagnetic coupling field between P1 and P2 layers. *H*_ex_ is the exchange coupling field between P1 and IrMn. By minimizing the total free energy, the magnetization angle *θ*_P1_ can be calculated.

**Figure 5:**
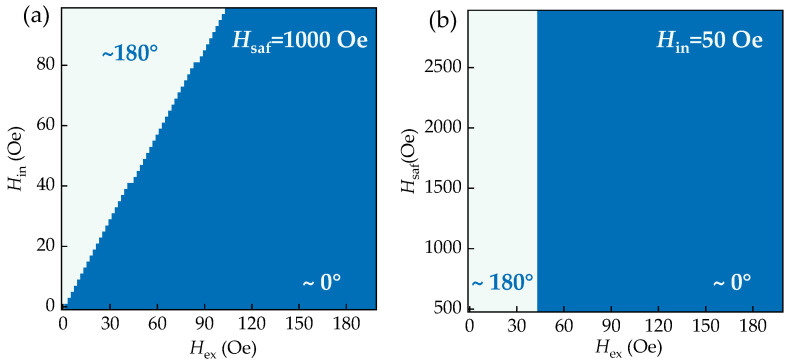
Heatmaps of the pinning direction: (**a**) the dependence of *θ*_P1_ on *H*_in_ and *H*_ex_; (**b**) the dependence of *θ*_P1_ on *H*_sat_ and *H*_ex_.

The expression of the total energy reveals that the pinning direction is determined by three coupling fields: *H*_ex_, *H*_saf_, and *H*_in_. These parameters are influenced by the thickness of the seed layer. Figure 5a depicts the pinning direction’s dependence on *H*_ex_ and *H*_in_, with *H*_saf_ set to 1000 Oe. The results show that the competition between these two coupling fields leads to two opposite possible pinning directions. Figure 5b verifies that the antiferromagnetic coupling in the SAF structure does not affect the pinning direction.

**Figure 6:**
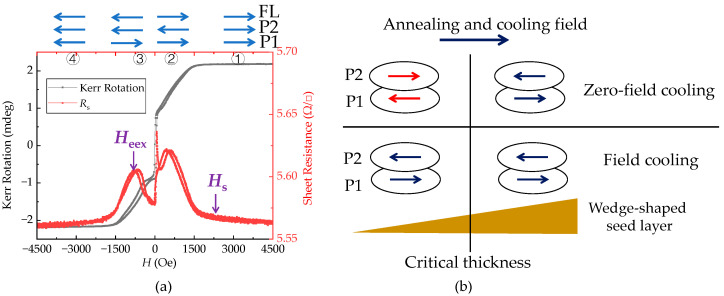
(**a**) Magnetization loops (black line) and Rs-H loops (red line) of the film with *t*_NiFeCr_ = 8.2 nm; (**b**) magnetization evolutions of P1 and P2 under different annealing processes.

A second annealing experiment is performed on this wafer using the same conditions as the first one, except that the 10,000 Oe field is kept during the cooling process. After this field cooling treatment, the magnetization loops and sheet resistance loops of the film are remeasured. Figure 6a shows the loops of the film with an 8.2 nm NiFeCr thickness after the second annealing. By comparing it with Figure 2b, it is found that the pinning direction is now realigned with the direction of the cooling field. Figure 6b summarizes the magnetization evolution of P1 and P2 under different annealing processes.

In the case of the zero-field cooling process, as the annealing temperature is near the blocking temperature of IrMn, the exchange coupling field *H*_ex_ generated at the IrMn/CoFe interface is small. *H*_ex_ is also related to the level of ordering of IrMn induced by the seed layer [29]. The interlayer coupling effect between the P2 and FL layers is ferromagnetic, which is mainly caused by the orange peel coupling from surface roughness. Because the total magnetization of P2 and FL is greater than that of P1, the P2 magnetization direction always tends to align with the annealing field direction. The P1 magnetization direction should be antiparallel to that of P2 due to the RKKY antiferromagnetic coupling. Meanwhile, the exchange coupling from the IrMn layer forces the magnetization direction of the P1 layer to go along with the annealing field direction. Therefore, in the film with a wedge-shaped seed layer, when the seed layer thickness exceeds a critical value, the exchange coupling effect dominates and the pinning direction is along the +*x* axis. The final state of the pinning direction may be along the +*x* (−*x*) direction when the exchange coupling effect is stronger (weaker) than that of the interlayer coupling effect.

In the case of a high field cooling process, the cooling-field strength is strong enough to saturate all the ferromagnetic layers, because the Zeeman energy of the external field is much larger than the RKKY coupling energy. After the removal of this field at room temperature, the P1 magnetization can maintain its direction since the exchange coupling field is induced along this direction.

## 5. Conclusions

The dependence of magnetic properties in the synthetic spin valve on the NiFeCr seed layer thickness has been studied. The results reveal that there is a critical seed layer thickness of 9.3 nm, above which the film exhibits good texture. The results highlight the crucial role of the seed layer in exchange bias effect and GMR performance. Meanwhile, after the zero-field cooling treatment, the pinning direction of the film has two possible orientations dictated by the competition between the exchange coupling and the interlayer coupling at high temperature. This phenomenon is verified by free energy model simulations and field-cooling experiments. The coupling mechanism for determining the pinning direction in synthetic spin valves has been clarified, which will benefit the design of GMR devices.

## Data Availability

Not applicable.
